# Mesenchymal Stem Cells for Perianal Crohn’s Disease

**DOI:** 10.3390/cells8070764

**Published:** 2019-07-23

**Authors:** Michele Carvello, Amy Lightner, Takayuki Yamamoto, Paulo Gustavo Kotze, Antonino Spinelli

**Affiliations:** 1 Colon and Rectal Surgery Unit, Humanitas Clinical and Research Center, 20089 Rozzano, Italy; 2 Department of Colon and Rectal Surgery, Cleveland Clinic, Cleveland, OH 44195, USA; 3 Inflammatory Bowel Disease Centre, Yokkaichi Hazu Medical Centre, Yokkaichi, Mie 510-0016, Japan; 4 Colorectal Surgery Unit, Cajuru University Hospital, Catholic University of Paraná, Curitiba 80215-901, Brazil; 5 Department of Biomedical Sciences, Humanitas University, 20089 Rozzano, Italy

**Keywords:** Crohn’s disease, stem cells, perianal, fistula

## Abstract

Perianal fistulizing Crohn’s disease (PFCD) is associated with significant morbidity and might negatively impact the quality of life of CD patients. In the last two decades, the management of PFCD has evolved in terms of the multidisciplinary approach involving gastroenterologists and colorectal surgeons. However, the highest fistula healing rates, even combining surgical and anti-TNF agents, reaches 50% of treated patients. More recently, the administration of mesenchymal stem cells (MSCs) have shown notable promising results in the treatment of PFCD. The aim of this review is to describe the rationale and the possible mechanism of action of MSC application for PFCD and the most recent results of randomized clinical trials. Furthermore, the unmet needs of the current administration process and the expected next steps to improve the outcomes will be addressed.

## 1. Introduction

Crohn’s disease (CD) is a chronic inflammatory disease of the gastrointestinal tract of unknown etiology, which continues to increase in incidence for unknown reasons, resulting in a significant burden to the healthcare system [1,2]. CD is characterized by persistent transmural inflammation anywhere along the gastrointestinal tract with a chronic remitting and relapsing behavior which leaves patients on chronic immunosuppression and recurrent operations to treat the disease symptoms, but both unable to cure the disease. Perianal CD, present in over 25% of patients with CD, is notoriously difficult to treat with available biologics and surgical procedures. These patients experience significant morbidity due to pain, persistent drainage, recurrent perianal sepsis, and ongoing need to access medical care resulting in increased costs [1,2] and impaired quality of life [2].

Unfortunately, 37% of patients with PFCD experience refractory disease [3]. As a result, patients cycle through numerous immunosuppressive medications that can have significant side effects, and more than 90% undergo multiple surgical interventions [4] putting them at risk of incontinence [5]. While up to 64% can achieve fistula healing with optimized tissue flaps [5], the majority of patients cannot have a flap constructed, and 40% of patients are left with active disease, facing a lifetime of debilitating morbidity or, alternatively, a proctectomy [6,7]. The current ineffective treatment paradigm leaves patients with incontinence, chronic narcotics, lost jobs, increased risk of opportunistic infection from biologics, increased incontinence from surgical intervention, and significantly impaired quality of life in thousands of patients. This dismal picture has spurred significant interest in investigating better treatment options that have the potential for improved efficacy without a risk of incontinence.

## 2. Background and Rationale

The intrinsic weakness of perianal tissue distressed by CD is perhaps the reason for the healing failure [8]. Previous studies have demonstrated that adult stem cells isolated from adipose tissue have the ability to differentiate into different subsets, including muscle specialized type [9]. Furthermore, it has been reported that cellular aspirate of human adipose tissue implies a subset of pluripotent mesodermal stem cells with a possible differentiation in myogenic, adipogenic, and chondrogenic types of cells [10]. Furthermore, human adipose-derived stem cells have emerged as crucial regulators of immune response. Indeed, human stem cells have been used in an experimental murine model of drug-induced colitis and sepsis, demonstrating, by systemic infusion, improvement of the severity of colitis and amelioration of sepsis [11]. Moreover, a reduction of the inflammatory infiltrate and downregulation of inflammatory mediators was found in target organs [12]. This evidence has driven the focus on clinical stem cells application in the overhaul of damaged tissues [13,14]. The successful use of mesenchymal stem cells (MSCs) for the treatment of a refractory rectovaginal fistula in the setting of CD was first reported in 2003 by Garcia-Olmo et al. [15]. The same group pioneered the first phase I trial in 2005 [16]. In this trial, addressing safety and feasibility of the procedure, five patients with perianal Crohn’s disease were initially treated with autologous stem cell injection along the perianal fistula. These promising results generated a wave of phase I [17,18,19,20,21] and phase II [22,23] to study the safety and efficacy of using MSCs to treat perianal CD. Afterwards, Garcia-Olmo reported fistula healing in 70% of patients treated with expanded adipose-derived stem cells in the phase II randomized clinical trial [24]. Despite the heterogeneity in protocols using allogeneic [17,19,21,22] or autologous MSCs [16,17,18,20,23,25] derived from both bone marrow [21,25] or adipose tissue [15,16,19,20,22], administered at various doses, delivered as a singular or repeat injection, delivered with [16,19,22] or without scaffolding [21,26], the results of all completed trials have been encouraging with regard to both safety and efficacy.

## 3. Mechanism of Action 

**Figure 1:**
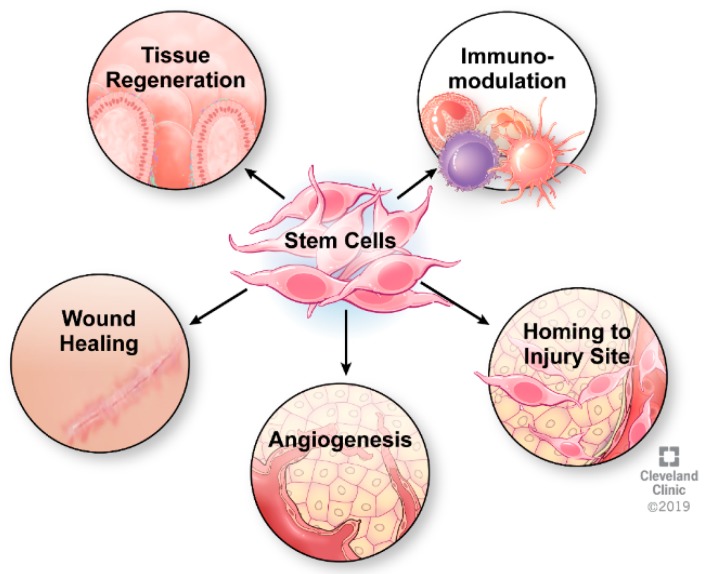
Mechanism of action of MSCs (courtesy of Cleveland Clinic, with permission).

While the exact mechanism of mesenchymal stem cells in treating Crohn’s disease remains unknown, it is well established that MSCs exist in almost all tissues [27,28,29] and are believed to reduce exacerbated inflammation due to their intrinsic immunomodulatory properties. Recently, success of MSCs in treating severe inflammatory disorders, such as graft-versus-host disease (GvHD) [30,31], systemic lupus erythematosus [32], myocardial infarction [33], multiple sclerosis [34], and Crohn’s disease (CD) [16], has highlighted the therapeutic benefit of the immunomodulatory characteristics of MSCs [35,36,37]. These immunomodulatory properties are carried out by three important steps: 1) migration to sites of active inflammation or tissue injury [38], 2) secretion of anti-inflammatory molecules like Interleukin-10 (IL-10), HGF, TGFβ1 [39], and Indoleamine 2,3-dioxygenase (IDO) [40], and 3) paracrine signaling to nearby cells to maintain the local anti-inflammatory environment [41,42] (Figure 1). By influencing cytokine secretion profiles [43], MSCs can modulate the function of various immune cell types including lymphocytes, dendritic cells, and macrophages [44]. Significant and specific to CD is the ability of MSCs to upregulate a CD4^+^ T cell subset of regulatory T cells (Tregs), a cell type known to be deficient in CD [28,45]. It has been well established that the depletion of Treg cells and imbalance of Treg to T effector cells play a key role in the pathogenesis of CD [46,47]. Therefore, MSCs’ ability to upregulate Treg cells, migrate to sites of inflammation [48], and dampen immune responses underscores the escalating interest in using MSCs to treat CD [49,50,51,52,53].

The mechanism of action of MSCs in Crohn’s disease has not yet been clarified in human studies. However, preclinical studies have shown an immunomodulatory effect of MSCs that is expressed by the inhibition of T cell function/proliferation as well as increased in regulatory T cells. The abovementioned effect is mediated by the induction of indoleamine 2,3 dioxygenase.

The immunosuppressive effect of MSCs in Crohn’s disease has been investigated in the ex vivo setting [54]. Indeed, the immunosuppressive effect on Crohn’s disease patients’ T cells was shown to be completely eliminated when blocking indoleamine 2,3 dioxygenase in cocultured plates. Furthermore, by using a semipermeable membrane to inhibit the contact between MSCs and T cells, the immunosuppressive effect was dampened, testifying to the need of cell-to-cell contact to express MSC function. Finally, no effects of mesenchymal stem cells were observed when T cells from control patients were cocultured with MSCs.

Notably, it has been reported that MSCs isolated from CD patients are functionally analogous to those of healthy individuals. Indeed, no differences were found in terms of phenotype, in vitro growth kinetics, and response to IFNγ. The immunomodulatory effect on T cell proliferation via indoleamin2.3 dioxygenase mechanism was not different between the two populations [55]. These findings could open the possibility of using native CD patients’ stem cells or drug targeting of native MSCs.

Interestingly we are uncertain whether bone-marrow-derived MSCs versus adipose-derived MSCs offer a better therapeutic approach; both have been studied independently for the treatment of perianal Crohn’s disease, but they have never been compared side by side in a clinical trial. In vitro, adipose-derived MSCs are clearly different than bone-marrow-derived, despite their similarities in cell surface expression markers [56]. Adipose MSCs replicate faster and proliferate longer in culture. In addition, the two cell types seem to differentiate along their lineages, toward an adipocyte versus an osteogenic capacity [56]. Adipose-derived MSCs have also been shown to have higher levels of secretion of cytokines that have been implicated in the immunomodulatory modes of action, including interleukin-6 and transforming growth factor-β1, and may have more potent immunomodulatory effects compared to bone marrow MSCs [57]. However, how this translates into clinical efficacy within clinical trials with Crohns’ disease remains unknown.

In addition, there is significant donor-to-donor variation in MSC function, and we still do not have a thorough understanding of who is an optimal donor. It is likely that different donors will be optimal for different diseases based on specific characteristics. For example, literature reports that older age significantly impacts proliferation and viability of MSCs [58], and that the female sex may improve the therapeutic effects of bone-marrow-derived MSCs via their increased anti-inflammatory properties [59]. Specific to CD is the recent evidence demonstrating that MSCs harvested from patients with CD exhibit reduced immunosuppressive capabilities when compared to MSCs from healthy donors [60].

To date, little is known about MSC behavior inside the injection site. Indeed, the pharmacokinetics studies are precluded because of the intended administration method (direct injection into the injured tissue). Animal model studies have demonstrated (after specimen retrieval and histopathology examination) that MSCs are able to reside in the injection site for a certain amount of time. Furthermore, their migration to the injury area has been shown. In order to address in detail the behavior of MSCs implanted in preclinical models and their impact on the site of application, labeling and tracking methods have been explored [61].

## 4. Application and Results of MSCs in Perianal Fistulizing Crohn’s Disease

Indications for the use of MSCs in perianal CD are mostly concentrated in fistulas. This is described in the label of the commercially approved product available in Europe (Alofisel^TM^, Darvadstrocel, Takeda Pharma A/S, Taastrup, Denmark). According to the label, the product is indicated for treatment of complex perianal fistulas in adult patients with nonactive/mildly active luminal CD, when fistulas have shown an inadequate response to at least one conventional or biologic therapy [62,63]. Alofisel^TM^ is composed of human allogeneic mesenchymal adult stem cells from adipose tissue (expanded adipose stem cells—eASCs). Cells are extracted from subdermal adipose tissue by liposuction, from healthy adult donors, and are subsequently expanded in laboratory facilities [62]. The adipose tissue obtained is then digested with type 1 collagenase to extract the MSCs, which are then separated by centrifuge. The cells are then expanded using cell culture techniques and harvested and cryopreserved. After plating, MSCs adhere to the plastic culture plates and are expanded under in vitro conditions. The culture medium has to be periodically changed until the cells reach 95% confluence. To require duplication, the expansion is performed without antibiotics. After detachment with trypsin/EDTA, the cells are collected and centrifuged. The product contains 4 vials of 6 mL solution which contains 30 million eASCs each, resulting in a total of 120 million cells, what corresponds to a concentration of 5 million cells per mL. Vials need to be kept at a temperature between 15 °C and 25 °C. Suspension of cells are settled in the bottom of the vial in a sediment form. After resuspension, the solution becomes a white/yellowish homogeneous suspension which can be injected in the patients. The preparation and preservation process, including immunologic profile screening and cell growth kinetics, is performed according to guidelines of the European Medicines Agency Committee for Advanced Therapies [64].

MSCs are generally well tolerated by the host patient. Due to the absence of HLA class II antigen, allogeneic MSCs retain an immunological privilege and are protected from innate and adaptive immunity [65]. Indeed, the development of donor-specific antibodies in treated patients has not been associated with immune response or treatment-related adverse events.

The product needs to be used after surgical conditioning of the fistula, with curettage of the track and closure of the internal opening with a stitch. Despite this fact, there is rationale for injection of MSCs in other situations. After commercial approval, indications for the use of stem cells in perianal CD will probably be explored further in other phenotypes, as rectovaginal fistulas or persistent ulcers, for example [21].

Most studies which evaluated the efficacy of MSCs in perianal CD had small sample sizes, which warranted wider clinical trials. Some of the available data were case reports, small case series, or single-arm small studies. The largest pivotal trial published to date which evaluated efficacy and safety of MSCs in perianal fistulas in CD was entitled the ADMIRE-CD (Adipose-Derived Mesenchymal Stem Cells for Induction of Remission in Perianal Fistulizing Crohn’s Disease) trial [22]. The trial was a randomized, double-blind, placebo-controlled study that tested Cx601, a 24 mL solution with 120 million expanded adipose-derived MSCs in CD fistulas. Each vial of the product had 30 million cells, and a total of 4 vials of the product was used in each case. The main inclusion criterion was patients with inactive or mildly active luminal CD (CDAI of 220 or less) with associated complex perianal fistulas. Patients with active proctitis, rectal stenosis, ileostomies, colostomies, and rectovaginal fistulas were excluded.

All patients had a previous surgical procedure under anesthesia, with curettage of the fistula tract(s) and seton placement if needed (two weeks before the injection of the drug). In the main surgical procedure, an unblinded surgeon injected the MSC compound or placebo saline solution (randomized in a 1:1 ratio) in the internal opening and close to the fistula tracts, after simple closure of the internal opening with stitches. The surgeon had to be unblinded as there are clear differences between the compound and saline solution in the prefilled syringes.

The main objective of the study was to analyze combined remission (clinical closure of all treated external openings draining initially at baseline, and the absence of collections with more than 2 cm, confirmed by MRI) after 6 months (24 weeks), performed by blinded gastroenterologists and radiologists.

**Figure 2:**
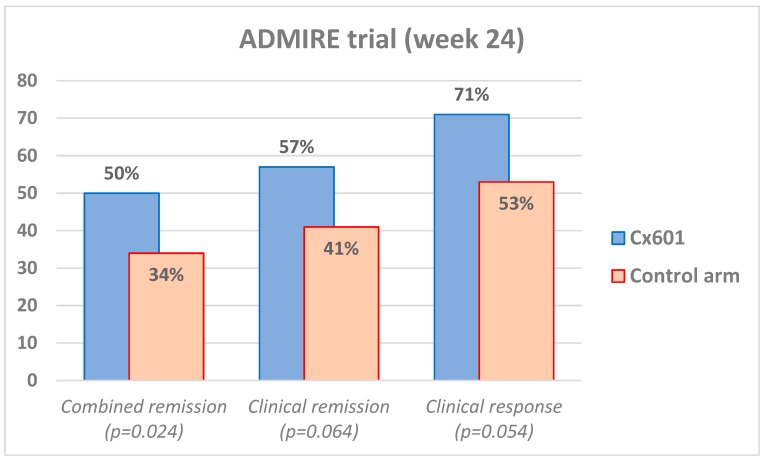
ADMIRE randomized trial results of efficacy at week 24.

107 patients had darvadstrocel injections and 105 had saline injections, as a control group. After 24 weeks, more patients in the compound group presented combined remission as compared to controls (53/107 [50%] versus 36/105 [34%], respectively, with a delta of 15.2% and 97.5% confidence interval 0.2–30.3; *p* = 0.024). Clinical remission alone (closure of 100% of external openings) was observed in 57% of the darvadstrocel/Cx601 patients as compared to 41% of placebo (*p* = 0.064). Clinical response was another secondary endpoint (closure of 50% of the fistula openings) and it was observed in 71% of the compound group as compared to 53% of placebo patients (*p* = 0.054). Results are illustrated in Figure 2. In terms of safety, a total of 66% (68/103) patients in the darvadstrocel group and 65% (66/102) in the control group had treatment-emergent adverse events, proctalgia, anal abscess, and nasopharyngitis being the most common. Treatment-related adverse effects were found in 17% in the study group as compared to 29% in placebo, mostly anal abscesses and proctalgia. Perianal abscesses occurred in 5% of the overall patients in both groups.

**Figure 3:**
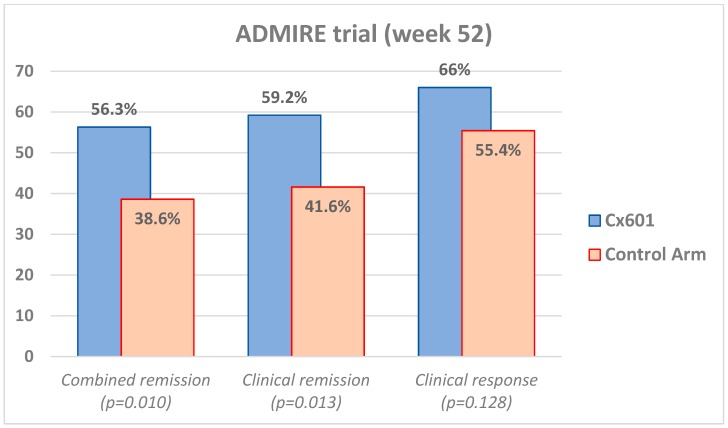
Long-term extension efficacy results of the ADMIRE randomized trial at week 52.

The long-term results (outcomes after 1 year—52 weeks) of the same trial were published in 2018 [26]. The patients from the ADMIRE-CD study were followed up to 52 weeks (1 year) and an additional MRI and a clinical evaluation were performed to check the same endpoints. Combined clinical and radiological remission was observed in 58/103 (56.3%) of the darvadstrocel/Cx601 patients, as compared to 39/101 (38.6%) in the control group, with a delta of 17.7 points, 95% CI 4.2–31.2; *p* = 0.010). Clinical remission (100% closure of baseline fistulas) after one year was observed in 59.2% in darvadstrocel/Cx601 and 41.6% in placebo groups (*p* = 0.013). Clinical response was observed in 66% and 55.4% in both groups, respectively, with *p* = 0.128. These findings are illustrated in Figure 3. Importantly, from the safety perspective, anal abscesses and fistulas were observed similarly between the groups in the one-year analysis (33% of the active group and 29.4% in the placebo group). Serious abscesses/fistulas were observed in only 6.8% and 4.9% in both groups, respectively. The rates of withdrawal of the study due to adverse events were low between the groups, 8.7% and 8.8%, respectively. No new safety signal in terms of new adverse events was observed in the additional 24 weeks of this long-term study.

A similar study is currently ongoing in the United States (Adult Allogeneic Expanded Adipose-Derived Stem Cells (eASC) for the Treatment of Complex Perianal Fistula(s) in Patients with Crohn’s Disease—ADMIRE-CD-II) to demonstrate efficacy for a future approval of darvadstrocel in America by the FDA (ADMIRE-CD-II trial, available in clinicaltrials.gov). In Europe, a postmarketing registry entitled INSPIRE (design and implementation aspects of a registry of complex perianal fistulas in Crohn’s disease patients treated with darvadstrocel) aims to establish a framework to capture real-world efficacy and safety data with this commercially available MSC product [61]. The registry is beginning to capture patients from different countries, and soon a more solid snapshot of patients with MSC local therapy will be available.

## 5. Safety

The risk of infection and tumor is of main concern with the use of MSCs. Indeed, the safety issue has yet to be fully addressed before the treatment is officially approved for its use on CD. While toxicity remains the most important limit for hematopoietic stem cell therapy in CD patients, MSCs have shown a relatively higher safety profile [66]. Serious adverse events (SAEs) requiring hospital admission are rare and might be more related to intrinsic disease activity. The studies that have been published to date indicate that administration of MSCs might prompt minor adverse events such as perianal sepsis. Indeed, a relatively high rate of perianal sepsis has been reported by phase I–II trials [16,17,20]. In the latest phase III trial published by Panés et al. [22], 68 patients (66%) in the treatment group and 66 (65%) in the control group developed AEs (adverse events), while SAEs (serious adverse events) were registered in 18 (17%) and in 14 (14%), respectively, the majority being anal abscess and proctalgia. In this study, the rates of AEs and SAEs were comparable to the control groups. Arguably, the side effects have been interpreted as not directly related to MSC administration but rather to the procedure adopted for the fistula closure or preconditioning before MSC administration. Indeed, a recent metanalysis of comparative studies has shown no significant difference in AEs and SAEs when comparing MSC and non-MSC groups of patients [67].

MSCs may show protumorigenic impact on cancers, by inducing neoplastic cell proliferation and promoting angiogenesis [68,69]. To date, there are no reported cases of neoplasm developed after MSC perianal treatment. However, long-term follow-up will clarify and strengthen also this safety aspect.

## 6. Future Perspectives

Several remaining questions in the treatment of perianal CD with MSCs remain to be addressed. One important issue is the presence of active proctitis during MSC administration. Proctitis is common in patients with perianal CD [70,71] but has remained an exclusion criteria from most clinical trials performed to date. Interestingly, the presence of proctitis may actually enhance the therapeutic benefit of MSCs, rather than hinder the treatment effect. However, this has yet to be determined. Moreover, even though more rare, rectovaginal and enterocutaneous fistula patients have been excluded so far from the trials.

Additional unanswered controversies include the ideal cell dosage and the optimal cellular delivery approach. In fact, no univocal cell dosage and administration procedure (direct injection, fibrin glue) has been consistently identified in the trials to date [72]. Rather, cell dosage has ranged from 20 to 120 million cells delivered, with variable protocols with regard to repeat injection, and various methods of delivery including direct injection, injection with fibrin glue, and delivery on a fistula plug. Further clinical trials comparing dose and delivery mechanism will help answer these questions.

In addition, once MSC administration becomes more widely available and utilized, comparative trials with standard therapy (including biologics and alternative surgical procedures) should be performed to validate the efficacy of this therapeutic approach.

In the future, it would be advantageous to also consider this treatment approach for luminal Crohn’s disease. The intestine is heavily populated with resident MSCs, as is the mesentery surrounding the intestine. The function of these MSCs as compared to healthy MSCs with immunomodulatory properties has not been studied, nor their contribution to the pathophysiology of the disease. Intestinal CD4+ T cells play a fundamental role in Crohn’s disease. In non-Crohn’s patients, there is a predominance of T cell regulatory mechanisms, which maintain intestinal homeostasis despite the daily presence of enormous microbial and antigenic load, and intestinal epithelial cells and antigen-presenting cells (APCs) which orchestrate mucosal innate immunity [73]. In Crohn’s disease, altered intestinal epithelial cells function as nonprofessional APCs that are unable to promote the expansion of T regulatory cells (Tregs). As such, they trigger a heightened/aberrant immune response [74]. Therefore, it is likely that resident MSCs are also aberrant and unable to fix this immune response. Of interest for future studies would be delivery of healthy donor MSCs to the intestine to see if this response could be changed and the population of T regulatory cells could be increased. This investigation, along with a better understanding of resident MSCs in Crohn’s versus normal patients, will serve to greatly expand MSCs’ therapeutic role in treating luminal Crohn’s disease.

## 7. Conclusions

The management of PFCD is controversial and actual available treatments present a relatively limited rate of success. MSC administration retains a high potential value in the treatment of PFCD. On the other hand, to date, the procedure is considered as an alternative to standard medical therapy and supplementary surgical procedures. Nonetheless, MSC administration is reported to be effective in inducing fistula healing. However, the mechanism promoting fistula healing is yet to be fully explored. Further studies are mandatory to determine the impact of MSC administration even in complex fistulas with multiple fistula tracts even in the presence of distal luminal disease. Additionally, the lack of fistula healing definition is, perhaps, the major barrier when results of trials are screened and compared to each other.
